# Out-of-Pocket Costs Among Patients With a New Cancer Diagnosis Enrolled in High-Deductible Health Plans vs Traditional Insurance

**DOI:** 10.1001/jamanetworkopen.2021.34282

**Published:** 2021-12-22

**Authors:** Sue J. Fu, Liam Rose, Aaron J. Dawes, Lisa M. Knowlton, Kathryn J. Ruddy, Arden M. Morris

**Affiliations:** 1 Stanford-Surgery Policy, Improvement Research, and Education Center, Department of Surgery, Stanford University School of Medicine, Stanford, California; 2 Health Economics Resource Center, Veterans Affairs Palo Alto Health Care System, Menlo Park, California; 3 Department of Oncology, Mayo Clinic, Rochester, Minnesota

## Abstract

**Importance:**

The financial burden of a cancer diagnosis is increasing rapidly with advances in cancer care. Simultaneously, more individuals are enrolling in high-deductible health plans (HDHPs) vs traditional insurance than ever before.

**Objective:**

To characterize the out-of-pocket costs (OOPCs) of cancer care for individuals in HDHPs vs traditional insurance plans.

**Design, Setting, and Participants:**

This retrospective cohort study used the administrative claims data of a single national insurer in the US for 134 826 patients aged 18 to 63 years with a new diagnosis of breast, colorectal, lung, or other cancer from 2008 to 2018 with 24 months or more of continuous enrollment. Propensity score matching was performed to create comparator groups based on the presence or absence of an incident cancer diagnosis.

**Exposures:**

A new cancer diagnosis and enrollment in an HDHP vs a traditional health insurance plan.

**Main Outcomes and Measures:**

The primary outcome was OOPCs among individuals with breast, colon, lung, or all other types of cancer combined compared with those with no cancer diagnosis. A triple difference-in-differences analysis was performed to identify incremental OOPCs based on cancer diagnosis and enrollment in HDHPs vs traditional plans.

**Results:**

After propensity score matching, 134 826 patients remained in each of the cancer (73 572 women [55%]; median age, 53 years [IQR, 46-58 years]; 110 071 non-Hispanic White individuals [82%]) and noncancer (66 619 women [49%]; median age, 53 years [IQR, 46-59 years]; 105 023 non-Hispanic White individuals [78%]) cohorts. Compared with baseline costs of medical care among individuals without cancer, a breast cancer diagnosis was associated with the highest incremental OOPC ($714.68; 95% CI, $664.91-$764.45), followed by lung ($475.51; 95% CI, $340.16-$610.86), colorectal ($361.41; 95% CI, $294.34-$428.48), and all other types of cancer combined ($90.51; 95% CI, $74.22-$106.79). Based on the triple difference-in-differences analysis, compared with patients without cancer enrolled in HDHPs, those with breast cancer paid $1683.36 in additional yearly OOPCs (95% CI, $1576.66-$1790.07), those with colorectal cancer paid $1420.06 more (95% CI, $1232.31-$1607.80), those with lung cancer paid $467.25 more (95% CI, $130.13-$804.37), and those with other types of cancer paid $550.87 more (95% CI, $514.75-$586.99).

**Conclusions and Relevance:**

Patients with cancer and private insurance experienced sharp increases in OOPCs compared with those without cancer, which was amplified among those with HDHPs. These findings illustrate the degree to which HDHPs offer poorer protection than traditional insurance against unexpected health care expenses. Coupled with the increasing cost of cancer care, higher cost sharing in the form of increasing enrollment in HDHPs requires further research on the potential clinical consequences through delayed or foregone care.

## Key Points

**Question:**

What is the association of a new cancer diagnosis and enrollment in a high-deductible health plan with out-of-pocket costs for patients?

**Findings:**

In this cohort study using propensity score matching and triple difference-in-differences analysis, 134 826 patients with a new diagnosis of cancer and enrolled in a high-deductible health plan faced sharp increases in out-of-pocket costs relative to those with traditional health plans and those without cancer.

**Meaning:**

This study suggests that private health insurance plans (particularly those with high cost sharing) leave patients vulnerable to considerable financial burden after receiving a diagnosis of cancer.

## Teaser

This cohort study uses administrative claims data from a single national insurer in the US to characterize the out-of-pocket costs of cancer care for individuals in high-deductible health plans vs traditional insurance plans.

## Introduction

Cancer care expenses are increasing owing to advances in imaging, immunotherapy, and biologic pharmacotherapy in addition to improved prognosis, with a subsequent need for prolonged treatment and surveillance.^1,2^ These welcome improvements in cancer care, however, have placed large financial burdens on some patients, with potential clinical consequences in the form of foregone or delayed care.^3^ The term *financial toxicity*, now widespread in medicine, first arose in the clinical cancer literature to address the growing recognition that cancer care has both physical and economic consequences for patients.^4^ Since then, several studies have demonstrated the financially destabilizing effects of cancer on patients, even among those with insurance.

Although the Patient Protection and Affordable Care Act was able to reduce the out-of-pocket costs (OOPCs) of treatment through expanded Medicaid coverage, private insurance remains the most prevalent source of health care coverage for individuals in the US, covering 61% of the nonelderly adult population.^5^ Among those with private health insurance, high-deductible health plans (HDHPs), which decrease premiums but increase cost sharing, have proliferated substantially, increasing from coverage for 4% of workers in 2006 to 30% in 2019.^6^ In theory, higher cost-sharing requirements (ie, out-of-pocket spending by patients) incentivize patients to make more efficient health care decisions.^7^ In practice, HDHPs sometimes reduce health care use and costs at the expense of necessary preventive services, including cancer screening procedures.^8,9^ The combination of increasing cancer care expenses and cost-sharing plans may threaten to overwhelm the financial means of privately insured patients with a new cancer diagnosis.

To address the real-time financial implications of high cost-sharing HDHPs in cancer care, policy makers, clinicians, and patients need information about the broad financial burden of cancer. Previous studies, however, have focused on only specific cancer subtypes and have focused primarily on estimated costs or patient-reported costs. We therefore assessed the association between a new cancer diagnosis across cancer subtypes and OOPCs among adults with private insurance.

## Methods

### Study Design

We performed a cohort study using the Clinformatics Data Mart Database (OptumInsight). The Clinformatics Data Mart is a statistically deidentified database of administrative health claims for members of a large, national managed care company. The data set comprises information from more than 62 million beneficiaries with commercial or Medicare Advantage health plans from 2003 to 2020. This study was exempted from review by the Stanford institutional review board because the data were deidentified. This study followed the Strengthening the Reporting of Observational Studies in Epidemiology (STROBE) reporting guideline.

### Study Participants

We defined 2 distinct cohorts for comparison. The cancer cohort was identified based on the *International Classification of Diseases, Ninth Revision* and *International Statistical Classification of Diseases and Related Health Problems, Tenth Revision* codes for breast, colorectal, lung, and other types of cancer (eTable 1 in the Supplement) diagnosed from 2008 to 2018. Patients with diagnoses of benign and in situ neoplasms and those with more than 1 primary tumor of any origin were excluded from the study. The comparison cohort consisted of individuals with no cancer diagnoses who were randomly assigned an event date from 2008 to 2018 to serve as a comparison point. The study was limited to patients aged 18 to 63 years with 24 months or more of continuous enrollment (12 months or more of continuous enrollment both prior to and after the event date, defined as the date of cancer diagnosis or randomly assigned date for the matched comparison cohort). Patients with missing data on observations for any of the following covariates were also excluded from analysis: age, sex, race and ethnicity, Charlson Comorbidity Index (CCI), HDHP enrollment, and insurance product type (health maintenance organization, point-of-service plan, exclusive provider organization, preferred provider organization, and indemnity). High-deductible health plan status was defined by the threshold for HDHP per the US Internal Revenue Service (IRS) for each year for individuals and families,^10^ as well as including patients with a Health Savings Account because these financial tools are only available with an HDHP. To account for patients with an HDHP but without a Health Savings Account, we summed the total deductible for the patient and family members in the year of each patient’s event date. Patients who exceeded the IRS thresholds were also classified as having an HDHP.

### Study Variables

The primary outcome of interest was OOPCs, which were calculated as the per-patient sum of the deductible, copay, and coinsurance payments from medical and pharmacy claims. All costs were adjusted for inflation to December 2019 US dollar amounts using the Consumer Price Index. Out-of-pocket cost was the dependent variable, while cancer type (breast, colorectal, lung, other, or none) and the month relative to the event date were the key independent variables. These specific cancer types were included because they comprise most solid tumor diagnoses each year. We did not include prostate cancer as a specific type because active surveillance (as opposed to treatment) is an accepted management strategy.

### Statistical Analysis

We performed difference-in-differences analyses^11^ to assess (1) the associations between an incident cancer diagnosis and OOPCs and (2) the associations between an incident cancer diagnosis with HDHP enrollment and OOPCs. The difference-in-differences method allowed us to compare OOPCs with a cohort of patients without cancer (ie, to address concerns that changes in OOPCs may have been due to secular causes unrelated to cancer care).

First, to understand OOPCs across cancer types, we examined the difference in OOPCs over time among individuals with a diagnosis of cancer vs those without. We constructed fixed-effects regression models to compare patients with cancer and patients without cancer. The unit of analysis was at the person-month and person-year level, and we compared monthly and yearly OOPCs before and after each individual’s event date. All costs were adjusted for inflation to December 2019 US dollar amounts. The monthly or yearly OOPC was the dependent variable, while cancer type (breast, colorectal, lung, other, or none) and time relative to the event date were the independent variables. As a sensitivity analysis, we reran all analyses using random effects and mixed effects and noted no change in findings. We plotted results as event study figures to evaluate the assumption of parallel trends in the outcome before the event date.

Next, to assess the incremental association of HDHP enrollment vs traditional plan enrollment with OOPCs among patients with cancer relative to the comparison cohort, we performed a triple difference-in-differences analysis^12^ comparing OOPCs across a third dimension of HDHP vs traditional health plans. The triple difference-in-differences analysis provided an estimate of the added exposure of having an incident cancer diagnosis and being enrolled in an HDHP. Our main triple difference-in-differences model measured the associated difference in OOPCs with a 3-way interaction by cancer status (cancer vs no cancer), time (before vs after an event, where the event was the diagnosis date for patients with cancer and a randomly assigned date for patients without cancer), and HDHP status (HDHP vs traditional health plan enrollment).

To further reduce confounding risk in the analysis, we matched patients in the cancer and comparison cohorts on observable characteristics using propensity scores.^13,14^ The propensity scores were estimated by logistic regression, with likelihood of receiving a cancer diagnosis as the dependent variable. Age, sex, race and ethnicity, CCI, HDHP enrollment, insurance product type, state of residence, and interactions between age and HDHP as well as between age and CCI were the independent covariates based on a significance threshold of *P* < .20. Matching was performed using the nearest neighbor method with 0.001-width caliper and at a 1:1 ratio. We confirmed the balance among the covariates using standardized mean differences less than 0.1 and visually confirmed with distribution balance density function plots and Love plots (eFigure 1 in the Supplement). To confirm the robustness of our findings, we performed the analysis with exact matching and found similar results. We compared characteristics of traditional plan holders with HDHP enrollees within each matched cohort to assess baseline differences.

All statistical analyses were performed from June 1, 2020, to May 31, 2021, using Stata, version 16 (StataCorp LLC). All *P* values were from 2-sided tests, and results were deemed statistically significant at *P* ≤ .05.

## Results

**Figure 1.**
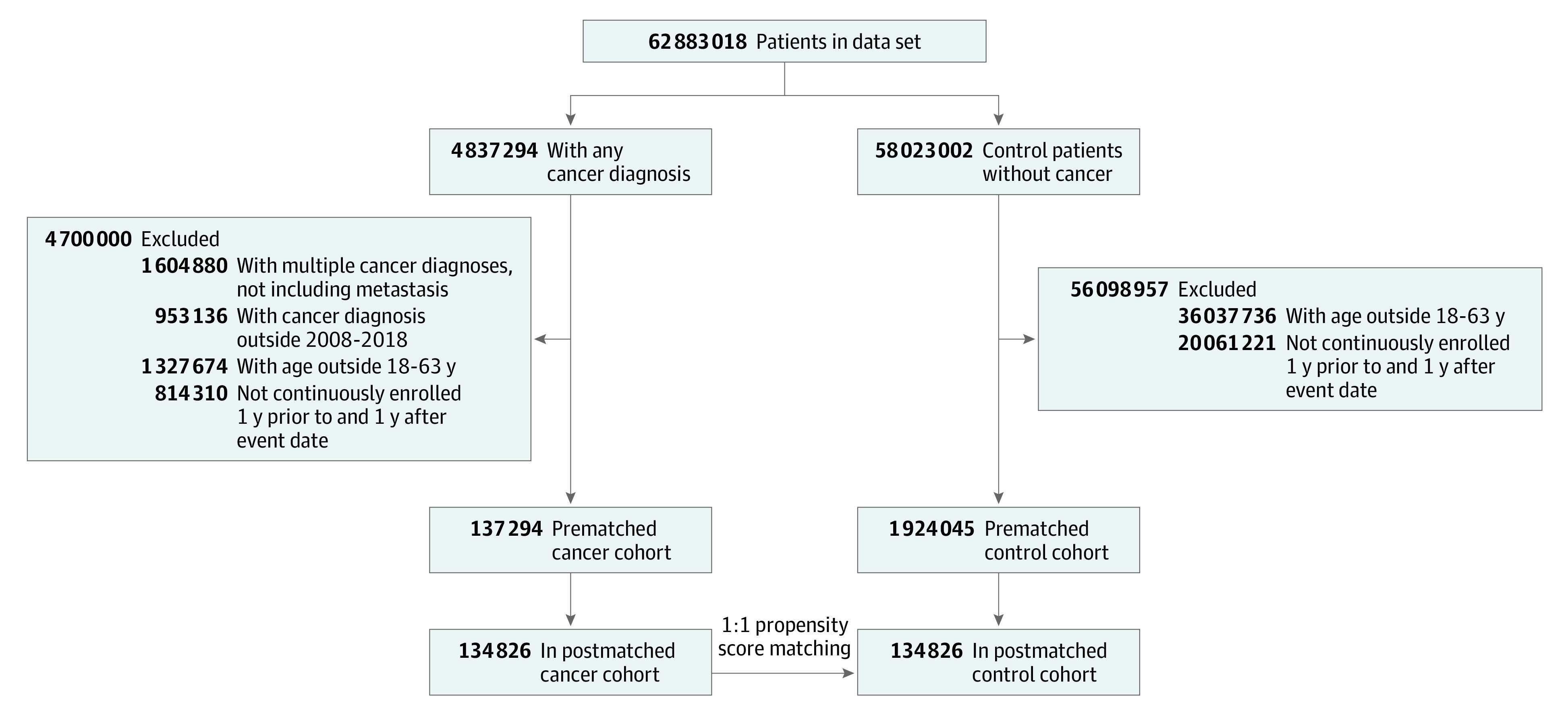
Diagram of Study Cohort Selection

A total of 137 294 patients from the single national insurer data set with a diagnosis of breast (n = 17 751), colorectal (n = 5012), lung (n = 1922), or other types of cancer (n = 112 609) and 1 924 045 control individuals from the single national insurer data set with no cancer diagnosis met the inclusion criteria. Prior to propensity score matching, patients with cancer were significantly older than comparison patients (median age, 53 years [IQR, 45-58 years] vs 42 years [IQR, 31-52 years]; *P* < .001), had more comorbid diseases (median CCI, 2 [IQR, 1-3] vs 0 [IQR, 0-1]; *P* < .001), and were more likely to be enrolled in an HDHP (49% [n = 67 264] vs 31% [n = 597 491]; *P* < .001; eTable 2 in the Supplement). After propensity score matching, 134 826 patients remained in each of the cancer (73 572 women [55%]; median age, 53 years [IQR, 46-58 years]; 110 071 non-Hispanic White individuals [82%]) and comparison (66 619 women [49%]; median age, 53 years [IQR, 46-59 years]; 105 023 non-Hispanic White individuals [78%]) cohorts for analysis (Figure 1). The postmatching distribution balance density function, Love plots, and standardized mean differences less than 0.1 indicated covariate balance for all variables (eFigure 1 in the Supplement).

Among the cancer cohort, 65 377 patients (48%) were enrolled in an HDHP, while 58 698 patients (44%) in the matched comparison cohort participated in an HDHP (Table 1). The median age was similar between patients enrolled in traditional plans and patients enrolled in HDHPs (patients with cancer, 52 years [IQR, 45-58 years] vs 54 years [IQR, 46-59 years]; comparison cohort, 53 years [IQR, 46-59 years] vs 54 years [IQR, 47-59 years]), as was the sex distribution (patients with cancer, 37 722 of 69 449 women [54%] vs 35 850 of 65 377 women [55%]; comparison cohort, 37 730 of 76 128 women [50%] vs 28 889 of 58 698 women [49%]), and median CCI (patients with cancer, 2 [IQR, 1-3] vs 2 [IQR, 1-3]; comparison cohort, 3 [IQR, 1-3] vs 3 [IQR, 1-3]). The race and ethnicity composition differed between the traditional plan enrollees and the HDHP enrollees, with more non-Hispanic White patients and fewer patients of socioeconomically disadvantaged racial and ethnic groups enrolling in HDHPs among both the cancer (54 713 of 69 449 non-Hispanic White patients in traditional plans [79%] vs 55 358 of 65 377 [85%] non-Hispanic White patients in HDHPs; *P* < .001) and comparison cohorts (57 417 of 76 128 [75%] non-Hispanic White patients in traditional plans vs 47 606 of 58 698 [81%] non-Hispanic White patients in HDHPs; *P* < .001).

**Table 1.** Baseline Characteristics of Patients With Traditional Insurance Plans vs HDHPs by Matched Cancer and Control Cohorts

Characteristic	Cancer cohort		Comparison cohort	
	Traditional plan (n = 69 449 [52%])	HDHP (n = 65 377 [48%])	Traditional plan (n = 76 128 [56%])	HDHP (n = 58 698 [44%])
Age, median (IQR)	52 (45-58)	54 (46-59)	53 (46-59)	54 (47-59)
CCI, median (IQR)	2 (1-3)	2 (1-3)	3 (1-3)	3 (1-3)
Sex, No. (%)				
Female	37 722 (54)	35 850 (55)	37 730 (50)	28 889 (49)
Male	31 727 (46)	29 527 (45)	38 398 (50)	29 809 (51)
Race and ethnicity, No. (%)				
Asian	2523 (4)	1192 (2)	2887 (4)	1119 (2)
Black	5312 (8)	4651 (7)	7681 (10)	5617 (10)
Hispanic	6901 (10)	4176 (6)	8143 (11)	4356 (7)
Non-Hispanic White	54 713 (79)	55 358 (85)	57 417 (75)	47 606 (81)
Cancer type				
Breast	7982 (12)	8317 (13)	NA	NA
Colorectal	2551 (4)	2319 (4)	NA	NA
Lung	966 (1)	880 (1)	NA	NA
Other	57 590 (83)	53 861 (82)	NA	NA
Insurance product, No. (%)				
HMO	17 312 (25)	3933 (6)	19 538 (26)	4010 (7)
POS plan	36 210 (52)	54 817 (84)	37 925 (50)	47 264 (81)
EPO	10 485 (15)	4039 (6)	11 366 (15)	4186 (7)
PPO	3713 (5)	2434 (4)	4303 (6)	3019 (5)
Indemnity	59 (0.08)	13 (0.02)	82 (0.1)	29 (0.05)
Other	1670 (2)	141 (0.2)	2914 (4)	190 (0.3)

Abbreviations: CCI, Charlson Comorbidity Index; EPO, exclusive provider organization; HDHP, high-deductible health plan; HMO, health maintenance organization; NA, not applicable; POS, point-of-service; PPO, preferred provider organization.

### OOPCs Associated With a Cancer Diagnosis

As anticipated, patients with cancer paid higher OOPCs during the months after diagnosis compared with patients without cancer after the random event date. Overall, increases in median monthly OOPCs among patients with cancer started 1 to 2 months prior to diagnosis, sharply peaked in the month of diagnosis, and decreased back to baseline more than 2 to 3 months after diagnosis (eFigure 2A in the Supplement). By contrast, yearly OOPCs among patients with cancer differed significantly depending on cancer type (eFigure 2B in the Supplement). Patients with breast cancer paid the highest difference in yearly OOPCs after the cancer diagnosis at $705.42 (95% CI, $0-$3027.19), followed by those with colorectal cancer ($330.13; 95% CI, −$25.53 to $2246.54), those with lung cancer ($271.17; 95% CI, −$276.19 to $1774.36), and those with other types of cancer ($220.34; 95% CI, −$66.25 to $1125.46).

### Difference-in-Differences Analysis

**Figure 2.**
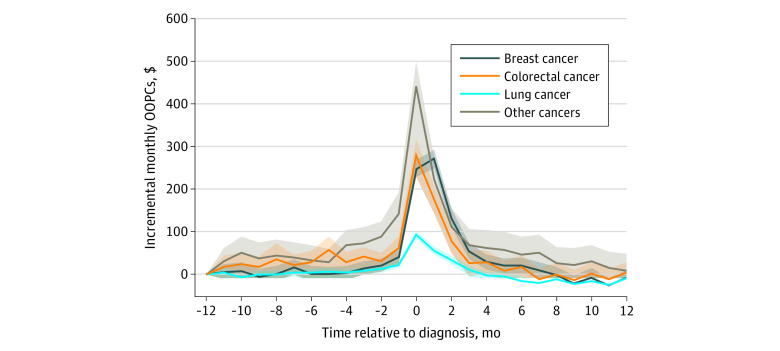
Estimates of Monthly and Yearly Out-of-Pocket Cost (OOPC) Difference in Differences Between Patients With Cancer and Patients Without Cancer The shaded areas indicate 95% CIs.

In the difference-in-differences analyses, we found that a cancer diagnosis was associated with sharp increases in additional monthly financial burden, centered around the month of diagnosis, compared with individuals without cancer (Figure 2). There were significant differences in baseline patterns of additional financial burden between types of cancer. Patients with lung cancer experienced the highest differences in OOPCs compared with patients without cancer relative to their baseline monthly OOPCs at 1 year prior to diagnosis, at an estimated monthly maximum of $439.80 (*P* < .001). Patients with breast, colorectal, or other types of cancer paid monthly OOPCs similar to those of patients without cancer until 1 month prior to diagnosis, when the monthly OOPCs increased, peaked in the diagnosis month or the month afterward ($270.50 for patients with breast cancer, $279.80 for patients with colorectal cancer, and $92.64 for patients with other types of cancer; *P* < .001), and remained statistically significantly elevated compared with patients without cancer until 2 to 4 months after diagnosis. On a yearly basis, a breast cancer diagnosis was associated with the highest additional OOPCs of $714.68 (95% CI, $664.91-$764.45) compared with patients without cancer, followed by lung cancer ($475.51; 95% CI, $340.16-$610.86), colorectal cancer ($361.41; 95% CI, $294.34-$428.48), and other types of cancer ($90.51; 95% CI, $74.22-$106.79) (Table 2).

**Table 2.** Yearly OOPC Differences

Cancer group	Difference in differences^a^		Triple difference in differences^b^	
	Incremental yearly OOPCs (95% CI), $	*P* value	Incremental yearly OOPCs (95% CI), $	*P* value
Control	1 [Reference]		1 [Reference]	
Breast	714.68 (664.91-764.45)	<.001	1683.36 (1576.66-1790.07)	<.001
Colorectal	361.41 (294.34-428.48)	<.001	1420.06 (1232.31-1607.80)	<.001
Lung	475.51 (340.16-610.86)	<.001	467.25 (130.13-804.37)	.007
Other	90.51 (74.22-106.79)	<.001	550.87 (514.75-586.99)	<.001

Abbreviation: OOPC, out-of-pocket cost.

^a^ Difference in differences in yearly OOPCs paid by patients with cancer relative to patients without cancer.

^b^ Triple difference in differences in yearly OOPCs paid by patients with cancer enrolled in high-deductible health plans relative to traditional health plans.

### OOPCs by Type of Health Insurance Plan

Among the comparison cohort of individuals without cancer, the median monthly OOPCs remained relatively stable during the 2-year study period; however, those without cancer and enrolled in HDHPs paid a median $63.88 (IQR, $16.80-$111.30) in monthly OOPCs compared with $30.45 (IQR, $0-$230.40; *P* < .001) paid by those in the comparison group with traditional plans (eFigure 3A in the Supplement). Patients with cancer enrolled in HDHPs paid significantly more in the year after their cancer diagnosis compared with patients with cancer enrolled in traditional plans, ranging up to $1975.99 (breast cancer), while the patients enrolled in a traditional plan paid only $160.00 (eFigure 3B in the Supplement).

### HDHPs and Cancer

**Figure 3.**
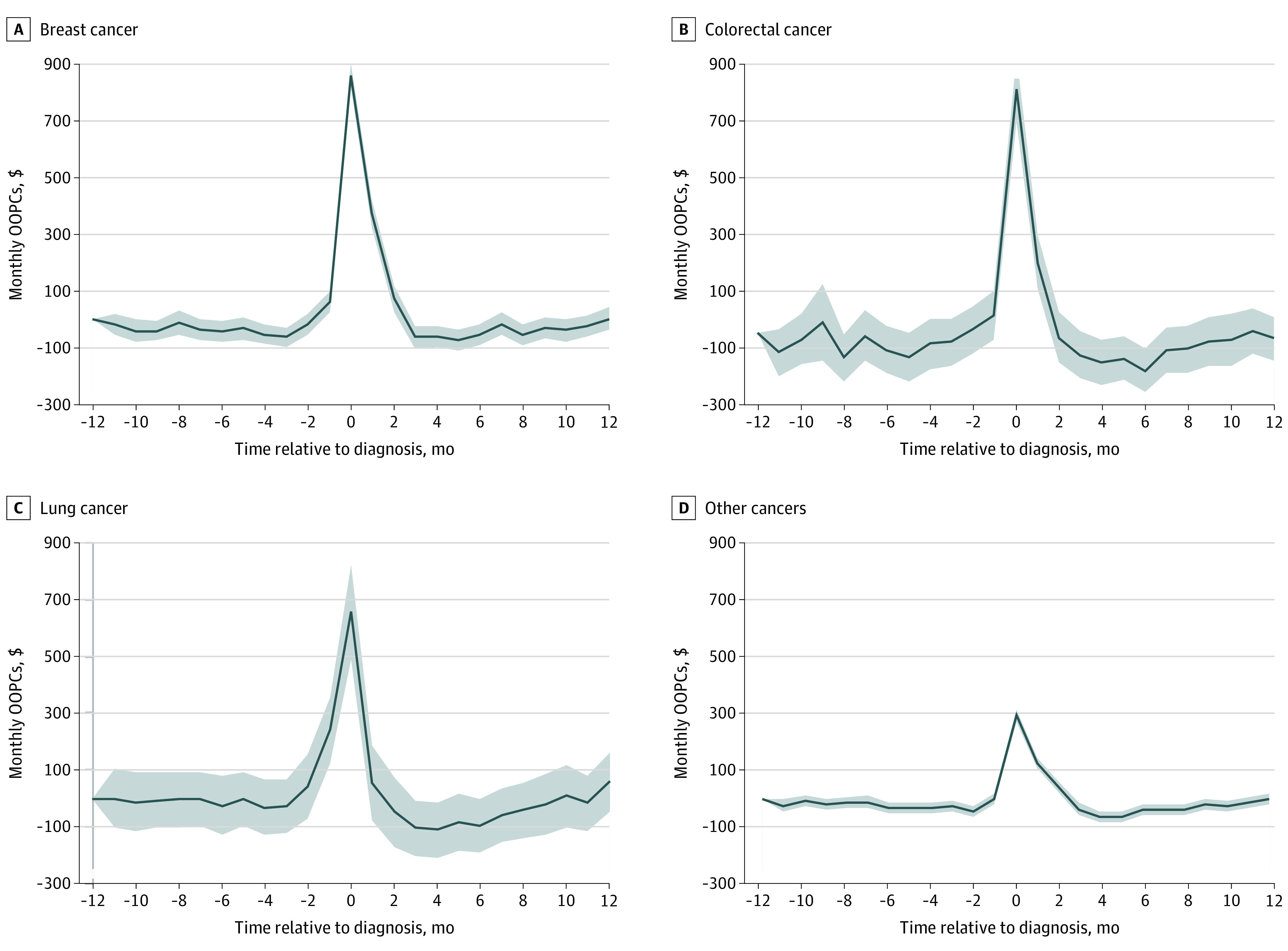
Estimates of Monthly and Yearly Out-of-Pocket Cost (OOPC) Triple Difference in Differences Among Patients With Cancer Relative to Patients Without Cancer, Between Those With High-Deductible Health Plans and Those With Traditional Health Plans The shaded areas indicate 95% CIs.

Next, we examined the incremental cost incurred by patients with cancer enrolled in HDHPs. The triple difference-in-differences analysis (Figure 3A) revealed that the incremental OOPCs associated with a cancer diagnosis and HDHP enrollment were substantial. Among patients with colorectal cancer, those enrolled in a HDHP paid $865.20 more (95% CI, $747.60-$982.90) in monthly OOPCs during the month of diagnosis than those enrolled in a traditional plan (*P* < .001). Among patients with breast cancer, those enrolled in an HDHP faced maximum monthly OOPCs of $860.00 more (95% CI, $814.40-$905.50) than those enrolled in a traditional health plan after their diagnosis (*P* < .001). Patients with lung cancer or other types of cancer who were enrolled in an HDHP also paid significantly more in monthly OOPCs at the time of diagnosis compared with their counterparts enrolled in a traditional plan (patients with lung cancer paid $655.40 more [95% CI, $490.40-$820.40] and patients with other types of cancer paid $292.30 more [95% CI, $272.90-$311.80]; *P* < .001).

Compared with patients without cancer enrolled in traditional plans, the incremental yearly OOPC estimates among patients with cancer enrolled in HDHPs was considerable (Figure 3B). Among patients enrolled in HDHPs, those with breast cancer paid $1683.36 (95% CI, $1576.66-$1790.07) more in yearly OOPCs, patients with colorectal cancer paid $1420.06 (95% CI, $1232.31-$1607.80) more, patients with lung cancer paid $467.25 (95% CI, $130.13-$804.37) more, and patients with other types of cancer paid $550.87 (95% CI, $514.75-$586.99) more.

## Discussion

Using a large claims data set, we found that insured patients with cancer experienced sharp increases in OOPCs compared with patients without cancer. Around the time of diagnosis, patients with cancer paid an additional $93 to $440 in monthly OOPCs compared with patients who did not have cancer. During the year after diagnosis, patients with cancer paid up to $715 more in yearly OOPCs than patients without cancer. Patients with cancer enrolled in an HDHP were subject to more extreme increases in OOPCs compared with their counterparts with traditional health plans, with monthly OOPCs peaking at $865.20 and accumulating to $1683 during the year after diagnosis.

To our knowledge, this is the first analysis of out-of-pocket financial burden across different types of cancer among a large, privately insured population using actual claims data. Previous studies on costs associated with a cancer diagnosis have been limited to patient-reported or estimated costs,^2,15,16,17,18,19^ which offer valuable insights but lack the capacity for verification. Patient self-reported outcomes suggest that nonelderly patients with cancer pay up to nearly $9000 in excess annual medical expenditures compared with patients without cancer, which represents publicly and privately insured patients as well as uninsured patients.^20,21^ These are not trivial amounts. To place these figures in context, 40% of US adults would not be able to pay an unexpected expense of $400 without selling possessions or borrowing money, and 61% of households would not be able to cover $1000 in unexpected expenses with savings.^22,23^ Patients with cancer face higher rates of bankruptcy and emotional distress associated with their financial status after their cancer diagnoses.^24,25^ Overall, our findings support previous studies that patients with cancer are subject to high financial burdens that may be associated with their clinical decision-making.^26,27^

High-deductible health plans were developed to address the perceived risk of the moral hazards of health care. It was believed that unnecessary care would be avoided by offsetting the financial responsibility of medical care to patients. Enrollment in plans with increased cost sharing has been shown to be associated with health care behaviors and access to care.^28,29,30,31,32,33,34^ The landmark RAND Health Insurance Experiment, which randomly assigned families to health plans with varying levels of cost sharing, showed that cost sharing lowered health care use and therefore reduced health care costs.^35^ However, patients also reduced their use of both appropriate and inappropriate services—and this was especially so for low-income populations made vulnerable owing to a lack of financial resources. Patients with breast cancer in HDHPs experienced delays in diagnostic imaging by a month, delays in early-stage breast cancer diagnosis up to 6 months, and chemotherapy initiation by more than 7 months compared with patients with traditional plans.^34^ High-deductible health plans may attract consumers with their low premiums; however, single employees enrolled in HDHPs contributed just $100 less to their annual premiums compared with those enrolled in traditional plans, and families enrolled in HDHPs paid nearly $1000 more in annual premiums relative to families enrolled in traditional plans.^36^

To better understand the implications of HDHPs among patients with cancer, future studies should assess the association of HDHPs with clinical outcomes, such as adherence to guideline-based treatments for cancer, and mortality as HDHPs continue to increase in popularity.^37,38^ From 2007 to 2017, OOPCs increased 15% in the top 1% of spenders with employer-sponsored health insurance.^39^ Our study found that HDHPs increased from 33% to 63% among patients with cancer with private insurance over 2008 to 2018, with an accompanying increase in median annual OOPCs after diagnosis from $577 to $1381. Overall, our findings indicate that underinsurance is increasing among individuals in the US, even those with private insurance, and that this has significant financial implications.^32,40,41,42^

### Limitations

As with other administrative claims analyses, this study has several limitations. First, while the Clinformatics Data Mart Database provides valuable demographic characteristics and claims data, it does not include patient symptoms, clinical data, or insights into their decisions to seek care, limiting the ability to draw conclusions on causality. Second, specific cancer types not examined explicitly in this analysis may have expensive new therapeutics that may have outsized effects on some patients that are not adequately captured in our broad averages. Third, while our results reveal the actual costs paid by patients for their health care, the true financial burden of care is likely higher. The costs captured in the data set are limited to deductible, coinsurance, and copay costs and do not include premiums, costs indirectly related to care (such as lost wages), or any costs incurred from out-of-network services. Relatedly, it is possible that the HDHP was misclassified. Our definition of an HDHP required the total annual deductible of each patient’s household to meet the minimum deductible threshold as defined by the IRS for both individuals and families. Among patients with cancer, observed total charges in the event year for patients with a cancer diagnosis were far in excess of deductible thresholds (median [SD] total charges, $43 200 [$686 768] for all cancers; eTable 3 in the Supplement), and deductibles often exceeded the annual deductible minimums (median [SD], $1447 [$9577] for all cancers). However, individuals without cancer may not use enough health care services to meet the minimum HDHP deductible threshold even if they do have an HDHP. We noted no temporal trends around the randomly assigned event date for individuals without cancer, making it unlikely that this type of misclassification biases our results (eFigure 2 in the Supplement), and we note that misclassification would make our estimates conservative.

Fourth, private insurance is covered largely by employers in the United States; therefore, continuous enrollment was necessary to establish baseline comorbidity and ensure the validity of cancer diagnosis codes. However, excluding patients who lose insurance within a year of their cancer diagnosis may introduce selection bias because these patients may be more debilitated by their disease and more likely to lose employment. In addition, a small portion of patients may have recurrent cancers not captured in the data. Furthermore, our study was limited to adult patients younger than 64 years because nearly all individuals gain Medicare eligibility at 65 years of age and would see a decrease in the OOPCs by private insurers after that age. Nonetheless, by focusing on individuals with private commercial insurance, we have demonstrated that even these individuals, with the most robust financial protections available in the United States, experience a high financial burden.

## Conclusions

Our findings illustrate the degree to which HDHPs offer poor protection against unexpected health expenses, such as those incurred by a cancer diagnosis. For many individuals in the United States, economic decision-making and medical decision-making are deeply intertwined. Financially vulnerable patients and their families continue to face painful decisions about whether to undergo high-cost cancer treatment or to delay or forego care. Policy makers, clinicians, and patients must consider the association of underinsurance with both the financial and physical well-being of all individuals.

## Notes

Supplement. **eTable 1.** *ICD-9* and *ICD-10* Cancer Codes **eTable 2.** Baseline Characteristics of Pre- and Post-Matched Cancer and Control Cohorts **eFigure 1.** Balance Tests After Propensity Score Matching **eFigure 2.** OOPC by Cancer Type **eFigure 3.** OOPC of HDHP vs Traditional Plan Enrollees by Cancer Type **eTable 3.** Deductibles and Charges During the Event Year
