# Supplementary material for: Out-of-Pocket Costs Among Patients With a New Cancer Diagnosis Enrolled in High-Deductible Health Plans vs Traditional Insurance
Source: JAMA Netw Open. 2021 Dec 22;4(12):e2134282. doi: 10.1001/jamanetworkopen.2021.34282 (PMC8696568; doi:10.1001/jamanetworkopen.2021.34282)
Supplement: Supplement. — eTable 1. ICD-9 and ICD-10 Cancer Codes eTable 2. Baseline Characteristics of Pre- and Post-Matched Cancer and Control Cohorts eFigure 1. Balance Tests After Propensity Score Matching eFigure 2. OOPC by Cancer Type eFigure 3. OOPC of HDHP vs Traditional Plan Enrollees by Cancer Type eTable 3. Deductibles and Charges During the Event Year [file jamanetwopen-e2134282-s001.pdf]

# Supplementary Online Content

Fu SJ, Rose L, Dawes AJ, Knowlton LM, Ruddy KJ, Morris AM. Out-of-pocket costs among patients with a new cancer diagnosis enrolled in high-deductible health plans vs traditional insurance. *JAMA Netw Open.* 2021;4(12):e2134282. doi:10.1001/jamanetworkopen.2021.34282

**eTable 1.** *ICD-9* and *ICD-10* Cancer Codes

**eTable 2.** Baseline Characteristics of Pre- and Post-Matched Cancer and Control Cohorts

**eFigure 1.** Balance Tests After Propensity Score Matching

**eFigure 2.** OOPC by Cancer Type

**eFigure 3.** OOPC of HDHP vs Traditional Plan Enrollees by Cancer Type

**eTable 3.** Deductibles and Charges During the Event Year

This supplementary material has been provided by the authors to give readers additional information about their work.

© 2021 Fu SJ et al. *JAMA Network Open.*

**eTable 1.** *ICD-9* and *ICD-10* Cancer Codes

| Cancer type | *ICD-9* | *ICD-10* |
|---|---|---|
| Breast | 174-175 | C50 |
| Colorectal | 153-154 | C18-C20 |
| Lung | 162 | C34 |
| Other | 140-152; 155-161; 163-209 | C00-C17; C21-C33; C35-C7A |

© 2021 Fu SJ et al. *JAMA Network Open.*

**eTable 2.** Baseline Characteristics of Pre- and Post-Matched Cancer and Control Cohorts

| | Pre-matched | | Post-matched | |
|---|---|---|---|---|
| | Cancer n=137,294 (7%) | Control n=1,924,045 (93%) | Cancer n=134,826 (50%) | Control n=134,826 (50%) |
| Age, median (IQR) | 53 (45-58) | 42 (31-52) | 53 (46-58) | 53 (46-59) |
| CCI, median (IQR) | 2 (1-3) | 0 (0-1) | 2 (1-3) | 3 (1-3) |
| Sex, n (%) | | | | |
| Female | 76,015 (55%) | 1,064,918 (55%) | 73,572 (55%) | 66,619 (49%) |
| Male | 61,279 (45%) | 859,127 (45%) | 61,254 (45%) | 68,207 (51% |
| Race/ethnicity, n (%) | | | | |
| NHW | 112,107 (82%) | 1,376,858 (72%) | 110,071 (81%) | 105,023 (78%) |
| Black | 9,994 (7%) | 194,209 (10%) | 9,963 (7%) | 13,298 (10%) |
| Hispanic | 11,304 (8%) | 249,463 (13%) | 11,077 (8%) | 12,499 (9%) |
| Asian | 3,889 (3%) | 103,515 (5%) | 3,715 (3%) | 4,006 (3%) |
| Insurance Product, n (%) | | | | |
| HMO | 21,515 (16%) | 261,793 (14%) | 21,245 (16%) | 23,548 (17%) |
| POS | 93,021 (68%) | 1,341,410 (70%) | 91,027 (68%) | 85,189 (63%) |
| EPO | 14,707 (11%) | 221,699 (12%) | 14,524 (11%) | 15,552 (12%) |
| PPO | 6,164 (4%) | 78,511 (4%) | 6,147 (5%) | 7,322 (5%) |
| Indemnity | 72 (0%) | 473 (0%) | 72 (0%) | 111 (0%) |
| Other | 1,815 (1%) | 20,159 (1%) | 1,811 (1%) | 3,104 (2%) |
| HDHP, n (%) | 67,264 (49%) | 597,491 (31%) | 65,377 (48%) | 58,698 (44%) |

Abbreviations: n=number; IQR=interquartile range; CCI=Charlson comorbidity index; NHW=non-Hispanic White; HMO=health maintenance organization; POS=point-of-service; EPO=exclusive provider organization; PPO=preferred provider organization; HDHP=high deductible health plan

© 2021 Fu SJ et al. *JAMA Network Open.*

**eFigure 1.** Balance Tests After Propensity Score Matching

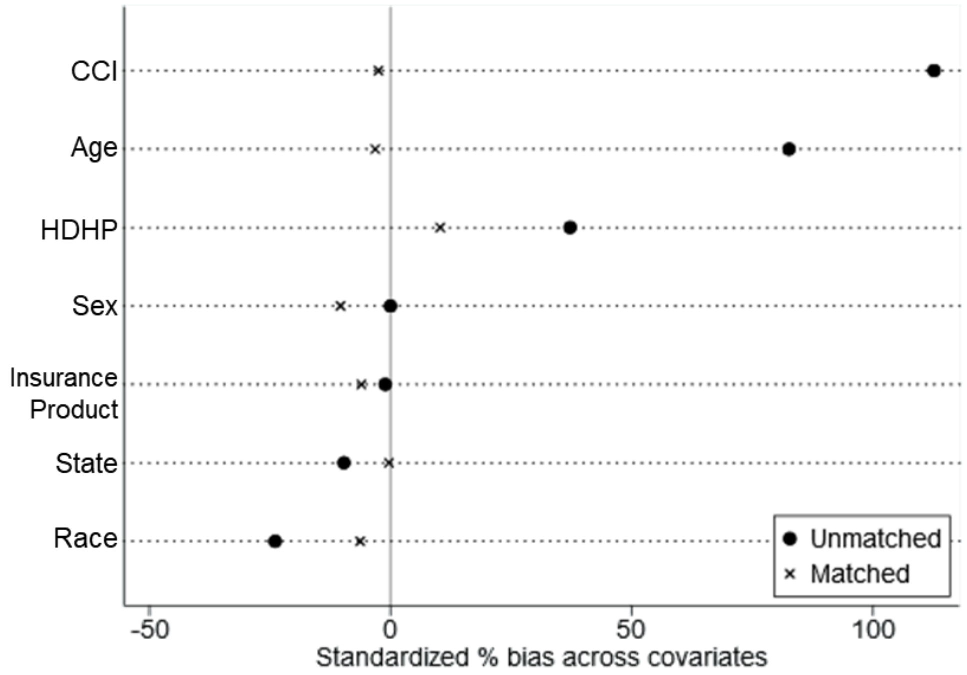


© 2021 Fu SJ et al. *JAMA Network Open.*

**eFigure 2.** OOPC by Cancer Type

A. Median Monthly OOPC by cancer type relative to event

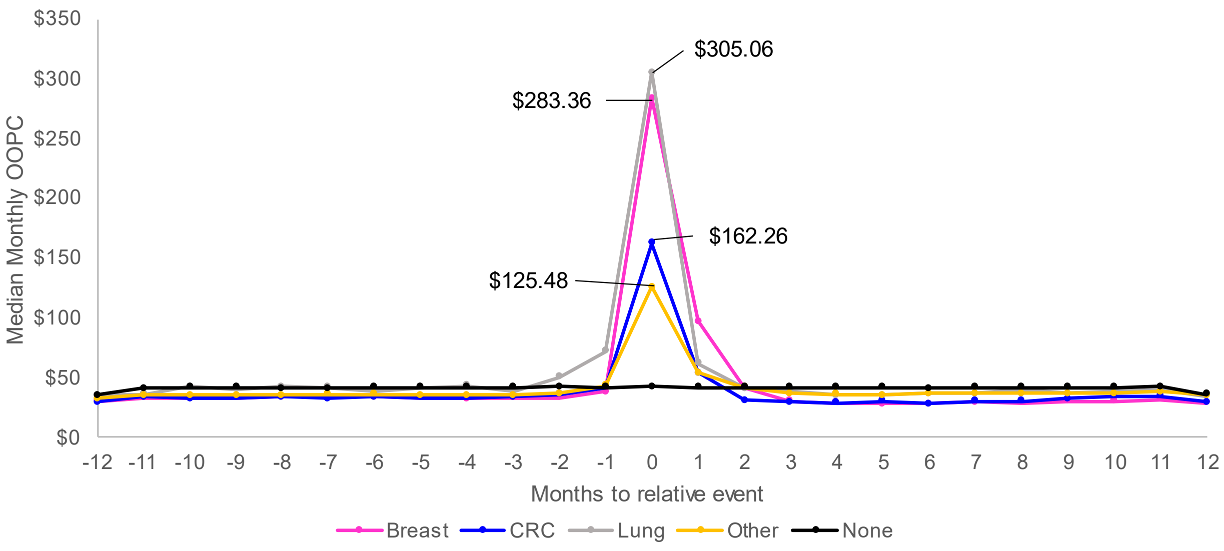


B. Median Yearly OOPC by cancer type relative to event

| Median yearly OOPC (IQR) | Pre-event | Post-event | Difference |
|---|---|---|---|
| Breast | $477.71 ($108.20-1,435.90) | $1,823.59 ($419.96-4,125.66) | $705.42 ($0-3,027.19) |
| Colorectal | $365.20 ($81.45-1,282.75) | $1,193.85 ($248.95-3,346.63) | $330.13 (-25.53-2,246.54) |
| Lung | $885.94 ($229.00-2,324.71) | $1,649.16 ($473.99-3,399.16) | $271.17 (-$276.19-1,774.36) |
| Other Cancer | $348.89 ($92.25-1,131.91) | $838.55 ($276.54-1,998.87) | $220.34 (-$66.25-1,125.46) |
| Control | $534.64 ($159.70-1,501.15) | $682.62 ($198.60-1,819.50) | $21.60 (-$363.97-712.05) |

*Comparison of yearly medianOOPC difference amongst cancer type
Abbreviations: OOPC=out-of-pocket costs; IQR=interquartile range; HDHP=high-deductible health plan

© 2021 Fu SJ et al. *JAMA Network Open.*

**eFigure 3.** OOPC of HDHP vs Traditional Plan Enrollees by Cancer Type

A. Median monthly OOPC of HDHP vs traditional plan enrollees by cancer type

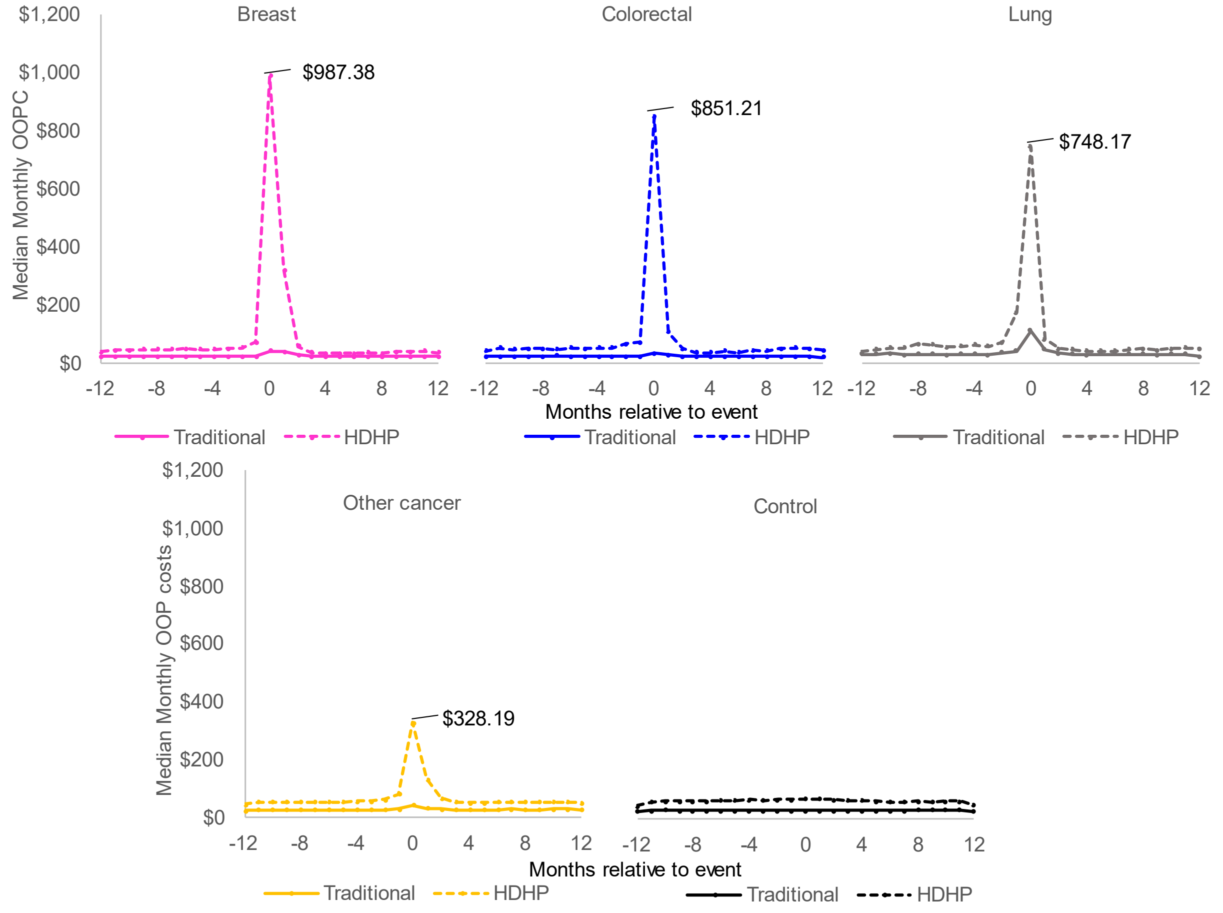


B. Median Yearly OOPC of HDHP vs traditional plan enrollees by cancer type

| Median Yearly OOPC (IQR) | Pre-event | | Post-event | | Difference | |
|---|---|---|---|---|---|---|
| | Traditional | HDHP | Traditional | HDHP | Traditional | HDHP |
| Breast | $180.00 ($33.60-604.26) | $1,023.49 ($355.69-2,218.74) | $542.96 ($107.70-1,936.78) | $3,406.85 ($1,673.59-5,760.61) | $160.00 ($0-1,250.16) | $1,975.99 ($155.03-4,508.38) |
| CRC | $150.82 ($23.80-538.41) | $897.71 ($252.76-2,220.14) | $349.04 ($74.55-1,250.52) | $2,815.79 ($1,277.81-5,003.11) | $65.54 (-$14.63-698.81) | $1,452.14 (-$93.34-3,900.41) |
| Lung | $413.13 ($102.08-1,254.80) | $1,737.22 ($725.73-3,264.17) | $782.60 ($205.36-2,257.93) | $2,568.76 ($1,282.30-4,518.74) | $133.02 (-$67.26-$1,055.20) | $644.99 (-$786.23-2,644.62) |
| Other cancer | $170.01 ($47.20-502.54) | $754.25 ($248.50-$1,935.75) | $347.28 ($125.27-843.84) | $1,670.34 ($887.42-3,003.66) | $81.20 (-$33.50-433.26) | $732.37 (-$210.66-1,938.67) |
| Control | $270.98 ($78.90-746.74) | $1,161.35 ($478.50-2,456.24) | $342.51 ($97.94-982.96) | $1,372.99 ($595.33-2,793.19) | $5.55 (-$168.6-376.92) | $103.84 (-$846.77-1,293.97) |

Abbreviations: OOPC=out-of-pocket costs; IQR=interquartile range; CRC=colorectal; HDHP=high-deductible health plan

© 2021 Fu SJ et al. *JAMA Network Open.*

**eTable 3.** Deductibles and Charges During the Event Year

| | All Cancers | Lung | Breast | CRC | Other | No Cancer | All Cancer - HSA | No Cancer - HSA |
|---|---|---|---|---|---|---|---|---|
| **Deductible** | | | | | | | | |
| Average | 5733 | 5817 | 6538 | 5763 | 5606 | 2740 | 16453 | 7743 |
| SD | 9577 | 9693 | 10440 | 9677 | 9425 | 6647 | 13883 | 11085 |
| Median | 1447 | 1250 | 1649 | 1238 | 1395 | 143 | 13013 | 2486 |
| | | | | | | | | |
| **Charges** | | | | | | | | |
| Average | 237176 | 542880 | 477434 | 426158 | 187000 | 77078 | | |
| SD | 686768 | 1261800 | 878016 | 904562 | 61435 | 364990 | | |
| Median | 43200 | 148728 | 158818 | 99539 | 35093 | 6567 | | |

CRC: Colorectal Cancer; HSA: Health Savings Account; SD: standard deviation.

© 2021 Fu SJ et al. *JAMA Network Open.*
